# Dosimetric Impact of Air Gaps in High-Dose-Rate Contact Interventional Radiotherapy (Modern Brachytherapy) for Non-Melanoma Skin Cancer of the Ear

**DOI:** 10.3390/jcm14217790

**Published:** 2025-11-03

**Authors:** Enrico Rosa, Bruno Fionda, Maria Vaccaro, Elisa Placidi, Valentina Lancellotta, Antonio Napolitano, Francesco Pastore, Francesca Greco, Pierpaolo Dragonetti, Maria Concetta La Milia, Gabriele Ciasca, Luca Tagliaferri, Marco De Spirito

**Affiliations:** 1 Unità Operativa Complessa Fisica per le Scienze della Vita, Dipartimento di Diagnostica per Immagini, Radioterapia Oncologica ed Ematologia, Fondazione Policlinico Universitario Agostino Gemelli IRCCS, 00168 Rome, Italy; 2 Department of Theoretical and Applied Sciences, Università Telematica eCampus, 22060 Novedrate, Italy; 3 Unità Operativa Complessa Degenze di Radioterapia Oncologica, Dipartimento di Diagnostica per Immagini e Radioterapia Oncologica, Fondazione Policlinico Universitario Agostino Gemelli IRCCS, 00168 Rome, Italy; 4 Medical Physics Unit, Bambino Gesù Children’s Hospital, 00165 Rome, Italy; 5 Dipartimento di Scienze Radiologiche ed Ematologiche, Università Cattolica del Sacro Cuore, 00168 Rome, Italy; 6 Sezione di Fisica, Università Cattolica del Sacro Cuore, 00168 Rome, Italy

**Keywords:** Contact High-Dose-Rate Interventional Radiotherapy, non-melanoma skin cancer, TG-43, TG-186

## Abstract

**Background/Objectives:** The anatomical complexity of the auricular region poses a unique challenge for contact interventional radiotherapy (IRT, modern brachytherapy), especially in maintaining close conformity between the applicator and skin surface. Air gaps can arise due to the irregular shape of the ear, potentially compromising dose coverage. This study evaluates the dosimetric impact of air gaps in HDR IRT for non-melanoma skin cancer (NMSC) of the ear. **Methods:** Ten patients treated with contact IRT using alginate as supporting material were retrospectively analyzed. Treatment plans were recalculated using both the TG-43 and the TG-186 formalism. CTV coverage and organ-at-risk dose parameters were evaluated within the two formalisms. **Results:** CTV coverage was comparable between algorithms (mean V95% 96.2% vs. 94.4%, V100% 89.6% vs. 86.7%, and V150% 2.6% vs. 2.5% for TG-43 vs. TG-186; *p* > 0.05), while the ipsilateral eye D2cc decreased from 4.0% (TG-43) to 3.2% (TG-186). In silico simulations showed that increasing air gaps reduced skin dose progressively (up to ~15% at 5 mm), whereas alginate thickness produced only a mild dose increase (<5%) across the tested range. Overall, small air pockets (<1 mm) did not substantially alter global dosimetric metrics, although local underdosage may occur at gap locations. **Conclusions:** This study underscores the importance of accounting for material heterogeneities and geometric uncertainties in anatomically complex regions through advanced dose calculation algorithms.

## 1. Introduction

Interventional radiotherapy (IRT, also known as modern brachytherapy) is a consolidated and effective technique for the treatment of non-melanoma skin cancers (NMSCs), offering highly localized dose delivery and excellent sparing of surrounding healthy tissues and organs at risk (OARs) [1]. Its high precision and ability to confine the dose to superficial layers make it particularly advantageous for lesions in anatomically and aesthetically delicate areas, such as the face and ears, where both tumor control and cosmetic preservation are critical. This technique is especially suitable for elderly or frail patients, providing an effective and minimally invasive alternative for lesions located in delicate anatomical sites [2].

Within this framework, contact High-Dose-Rate (HDR) IRT represents a widely used approach for superficial skin tumors, with treatment typically prescribed at shallow depths up to 5 mm from the skin surface. This technique requires accurate applicator positioning to ensure homogeneous dose delivery and reproducibility, especially in irregular or curved anatomical sites. Contact IRT achieves excellent local control while maintaining favorable cosmetic outcomes, particularly in visible areas such as the face and ears, where treatment-related disfigurement must be minimized [3].

Among these challenging sites, the auricular region presents unique technical difficulties due to its complex and irregular anatomy. The curved and thin surfaces of the ear often hinder stable and uniform contact between the applicator and the skin, leading to the formation of air gaps. These gaps can alter dose distribution, potentially resulting in underdosage of the clinical target volume (CTV) and compromising treatment efficacy. Although this issue is clinically relevant, the dosimetric consequences of air gaps in auricular IRT remain poorly characterized so far. More generally, the influence of air and supporting materials in contact IRT has been only marginally investigated across different dose calculation algorithms. In eyelid treatments, studies have specifically examined how the addition or omission of a bolus can influence dose distribution by reducing air gaps in the target area [4]. This concern has been addressed in other contact radiotherapy modalities such as intraoperative radiotherapy (IORT), where the presence of air gaps between the applicator and the target surface has been shown to significantly compromise dose homogeneity and reduce effective coverage [5]. Similarly, in HDR contact IRT, even small air pockets within 3D-printed applicators have been shown to distort dose delivery, leading to underdosage of the surface and compromising treatment accuracy, further emphasizing the need for detailed analysis of such effects in complex anatomical regions [6].

In such anatomically and technically complex regions, the involvement of a skilled multidisciplinary team is essential to ensure both the efficiency of the implantation procedure and the effectiveness of dose delivery, along with a comprehensive clinical evaluation. Depending on the complexity of the procedure and the anatomical site involved, different specialists may be required, including dermatologists for skin lesion assessment, otolaryngologists for procedures involving the ear or adjacent structures, in addition to the core team composed of the radiation oncologist, medical physicist, nurse and radiation technologist responsible for treatment planning, dose optimization, and technical execution [7].

To improve the interface between the applicator and the skin, alginate-based materials are frequently used during treatment setup. Owing to their biocompatibility, moldability, and ease of handling, alginates aid in stabilizing the applicator and enhancing surface conformity. While their primary role is mechanical, they may also influence dose distribution by acting as a physical bolus or modifying scattering conditions. However, their specific dosimetric contribution in this context has yet to be systematically evaluated. Recent developments in 3D printing have further expanded the possibilities for personalization in IRT. Alginate can be combined with 3D-printed patient-specific supports or templates derived from imaging data, enabling more precise and reproducible applicator placement. This integration enhances adaptability to individual anatomical features, minimizes air gaps, and improves dose conformity, representing a promising direction for the advancement of personalized contact IRT [8].

Despite these innovations, a systematic analysis of how the presence of air gaps of different thicknesses, whether or not mitigated by alginate, affects dose distribution and clinical parameters is still lacking for complex regions. Therefore, this study aimed to investigate the dosimetric impact of both alginate and air gaps in contact HDR-IRT for NMSCs of the ear. We evaluated their effect on dose distribution to the CTV and the ipsilateral eye and performed in silico simulations to quantify how variable air-gap and alginate thicknesses influence surface dose.

## 2. Materials and Methods

We conducted a retrospective review of our institutional electronic medical records to identify patients with histologically confirmed non-melanoma skin cancer (NMSC) who were treated with contact interstitial radiotherapy (IRT).

Regarding the clinical aspects, a total of 10 patients were included, with a mean age of 78 years (range: 62–90). The cohort was predominantly male (90%), with only one female patient (10%).

All procedures were performed with a remote afterloading system (Flexitron, Elekta^®^, Stockholm, Sweden) equipped with a high-dose-rate Iridium-192 (192Ir) source. The nominal initial activity at the time of source exchange was ~10 Ci, with decay-corrected source strength applied per institutional calibration records. The mean photon energy of 192Ir is ~0.38 MeV (multi-energy gamma spectrum), consistent with HDR IRT clinical use.

Treatment was administered in an exclusive setting in most cases (70%), while 30% underwent post-operative irradiation. Regarding exclusive treatment schedules, 40% of patients received 5 Gy × 8 fractions (twice daily), 30% received 3 Gy × 18 fractions; regarding the post-operative schedule it was 2.5 Gy × 20 fractions.

Laterality analysis showed that lesions were more frequently localized on the right ear (60%) compared to the left (40%). Histopathological examination revealed squamous cell carcinoma (SCC) in 70% of cases and basal cell carcinoma (BCC) in 30%.

At a median follow-up of 1 year, the local control rate was 90%. A summary of the main clinical findings, including age, gender, IRT prescription dose, clinical setting, histological type, and IRT implant information, is reported in Table 1.

Patients were included in the study if they had a histologically confirmed diagnosis of non-melanoma skin cancer (basal cell carcinoma or squamous cell carcinoma) and underwent contact interventional radiotherapy (IRT) using Freiburg flap applicators in combination with alginate to enhance surface conformity and applicator stability. Treatments were performed from January 2023 onward, and a minimum clinical follow-up of 12 months was required for inclusion.

All data were anonymized prior to analysis, and the study was conducted in accordance with institutional ethical standards.

**Figure 1:**
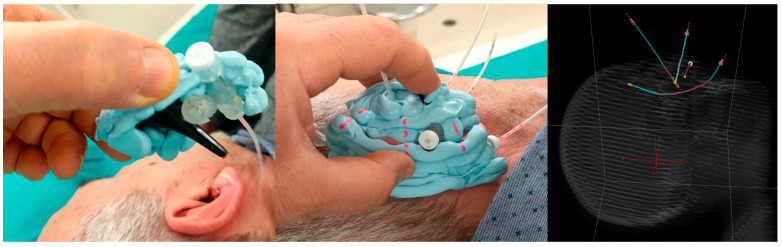
Three-dimensional CT reconstruction of the patient head and the Freiburg flap applicator adapted to the auricular region with hybrid implant configuration with the 4 catheters used (contact combined with intracavitary).

To illustrate the technical challenges associated with this anatomically complex region, a representative case is shown in Figure 1. To perform contact IRT in this patient, a Freiburg flap applicator was manually shaped and positioned along the auricular surface, with catheters inserted to follow the irregular geometry of the ear. This hybrid configuration, combining contact placement with elements of intracavitary adaptation, represents a practical strategy to ensure dose conformity in regions characterized by pronounced curvature and limited applicator support.

A computed tomography (CT) scan was acquired using a slice thickness of 1 mm for contact IRT treatments. For each patient, relevant anatomical structures were delineated in the Eclipse treatment planning system (Varian Medical Systems^®^, Inc., Palo Alto, CA, USA, v. 18), including the ipsilateral eye, the CTV, the alginate applied during treatment, the flaps used for implantation, and the external patient body. Catheter reconstruction and treatment planning were performed in the Oncentra Brachy v. 4.6 treatment planning system (Elekta^®^, Stockholm, Sweden), employing both the TG-43 and TG-186 dose calculation formalisms. TG-43 [9], the clinical standard, assumes a homogeneous water medium around the source and does not account for tissue heterogeneities or applicator materials. In contrast, a collapsed cone convolution algorithm (CCC, standard accuracy level—mean calculation time: 5 min and 42 s), implemented according to the TG-186 guidelines [10], incorporates a model-based approach that enables more accurate calculations by accounting for heterogeneities, including patient-specific materials and densities, thus allowing for more realistic dose estimation in anatomically complex areas such as the ear. The CCC algorithm implemented in Oncentra Brachy has been validated for research purposes in accordance with the AAPM TG-186 report through benchmark comparisons with Monte Carlo reference simulations provided by Elekta.

Material assignments were determined according to Hounsfield Unit (HU)–derived densities. The ipsilateral eye was contoured, whereas the lens was not. For TG-186 material assignment, the contoured eye ROI was mapped to the TPS “Eye Lens” material class with HU-based density scaling. The clinical target volume (CTV) and the external body were assigned “Soft Tissue” with HU-based density calculated from CT images. Catheters and the Freiburg flap were assigned “Plastic Water” with a fixed density of 1.014 g/cm^3^. The alginate structure was modeled using two different mass densities, 1.26 g/cm^3^ and 1.92 g/cm^3^, selected so that their arithmetic mean matched the nominal material density (~1.5 g/cm^3^). Dose calculations were performed independently for each density value, and the results were averaged to provide a representative dosimetric estimate consistent with the typical characteristics of clinical alginate formulations.

Dosimetric evaluation focused on parameters related to CTV coverage and OAR exposure between the two algorithms involved. For the CTV, the percentage volumes receiving 150%, 100%, and 95% of the prescribed dose (V150, V100, V95) were calculated. In addition, the dose to the ipsilateral eye, considered as the OAR, was assessed through the maximum dose delivered to the most exposed two cubic centimeters (D2cc), reported as a percentage of the prescribed dose.

Since the distributions of the analyzed parameters were not normal, as assessed by the Shapiro–Wilk test, the non-parametric Wilcoxon signed-rank test was applied to evaluate the statistical significance of the differences between TG-43 and TG-186 formalisms. A *p*-value < 0.05 was considered statistically significant.

**Figure 2:**
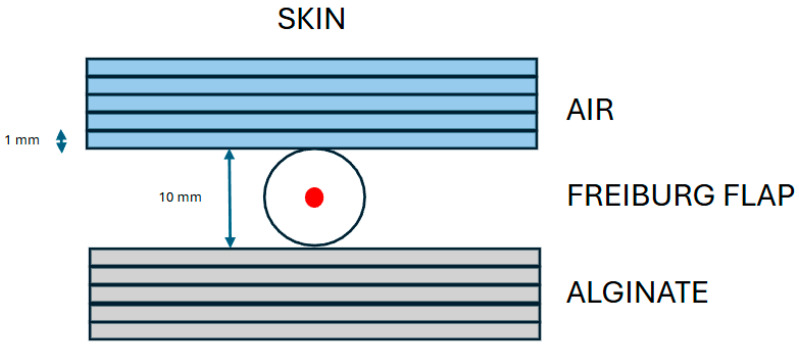
Schematic representation of the in silico simulation considering several thicknesses of air gaps and alginate.

Finally, to evaluate the individual contributions of air gaps and alginate on local dose distribution, an in silico simulation was performed by creating regions of interest (ROIs) representing air and alginate layers with variable thicknesses ranging from 0 to 5 mm for both materials. The catheter–skin reference geometry was fixed by the Freiburg flap at a 5 mm source-to-skin distance (baseline: 0 mm air, 0 mm alginate), as shown in a schematic representation in Figure 2. These layers were schematized as planar slabs positioned in a controlled geometric configuration: the air slab was placed between the source and the skin surface, while the alginate slab was positioned distally, beyond the source relative to the skin. A single fixed dwell position was activated in every simulation, and a theoretical treatment plan was generated so that the 150% isodose line tangentially reached the skin, based on TG-43 formalism. Subsequently, dose recalculations were performed using the CCC algorithm under the same computational conditions adopted for patient-specific dose calculations. For each configuration, the skin dose was calculated independently for the two densities, and the mean value of these results was used to represent the dosimetric effect of alginate in the analysis. The “skin dose” denotes the dose computed on the external patient surface (outer body contour) at the CTV–skin interface; this structure is distinct from the CTV contoured on CT. Finally, the differences in skin dose, reported as a percentage of the prescribed dose, were analyzed as a function of both air-gap thickness and alginate thickness.

## 3. Results

**Figure 3:**
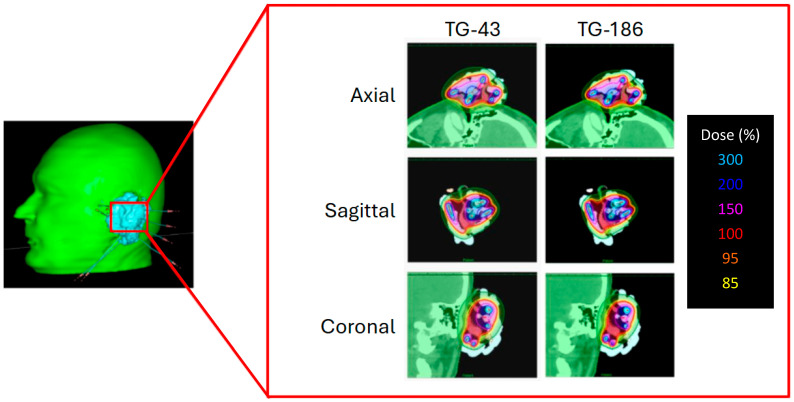
Three-dimensional reconstruction of the auricular implant with Freiburg flap applicator (**left**) and corresponding dose distributions calculated with TG-43 (**left column**) and TG-186 formalism (**right column**). Isodose curves are shown in axial, sagittal, and coronal planes.

To illustrate the effect of heterogeneity corrections, Figure 3 presents the dose distributions of a representative patient calculated with TG-43 and TG-186. The Freiburg flap applicator was adapted to the auricular region, and the resulting dose maps are displayed in axial, sagittal, and coronal views. Although TG-186 accounts for tissue and material heterogeneities, no appreciable differences in isodose coverage are observed when compared to TG-43.

Table 2 reports the dosimetric comparison performed between the TG-43 and TG-186 algorithms to evaluate potential differences in target coverage and OAR exposure. For TG-186, the reported values correspond to the mean of the two independent dose calculations performed with different alginate mass densities. The results are summarized in terms of V150%, V100%, and V95% of the CTV, as well as D2cc to the ipsilateral eye. Statistical analysis with the Wilcoxon signed-rank test revealed no significant differences for V150%, V100%, and V95% and D2cc of the ipsilateral eye when using TG-186.

**Figure 4:**
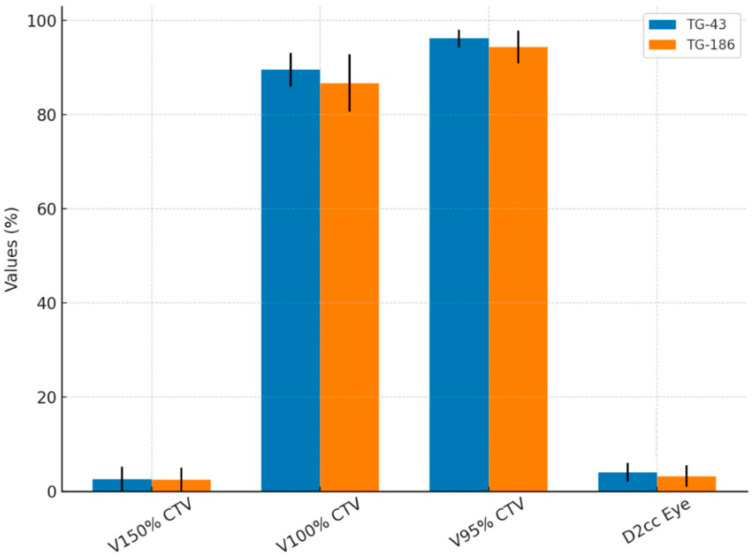
Histogram shows the comparison of coverage and dosimetric parameters between TG-43 and TG-186 algorithms.

To provide a clearer overview of the evaluation of the two algorithms in Table 2, Figure 4 summarizes the mean values and standard deviations of CTV coverage parameters (V150%, V100%, V95%) and D2cc to the ipsilateral eye obtained in the table.

**Figure 5:**
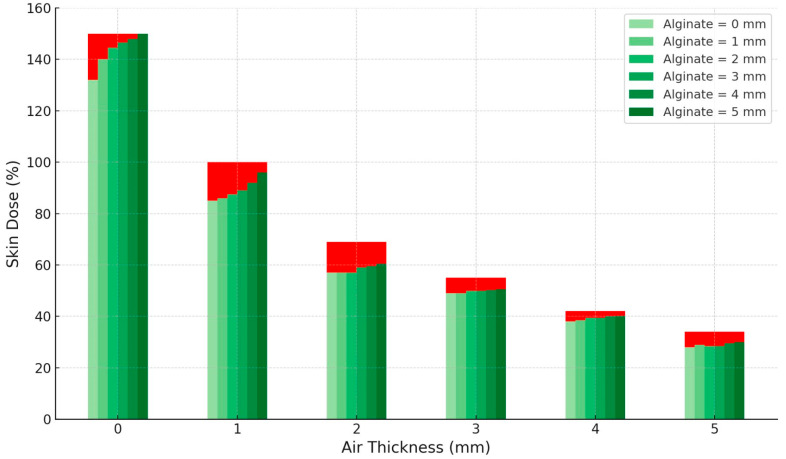
Variation in surface skin dose as a function of air-gap thickness and alginate thickness. The red bars represent the overall skin dose for several air-gap thicknesses, calculated using the TG-43 formalism, while the nested green bars within each column illustrate the effect of increasing alginate thickness (0–5 mm), calculated using the CCC algorithm implemented according to the TG-186 formalism.

CTV coverage metrics (V95%, V100%, V150%) are reported for clinical patient plans. Separately, Figure 5 illustrates the in silico sensitivity analysis of surface (skin) dose as a function of air-gap and alginate thickness, computed on the external contour at the CTV–skin interface under fixed dwell geometry. Increasing the air gap between the source and the skin surface resulted in a progressive and marked reduction in skin dose, reaching approximately 40% of the reference value at 5 mm. Conversely, increasing alginate thickness from 0 to 5 mm produced a mild compensatory rise in skin dose, up to about 5% within each air-gap condition. This indicates that while air gaps have a dominant dosimetric effect by reducing surface dose, the presence of alginate slightly mitigates this decrease through its scattering contribution.

Acute skin toxicity was evaluated according to the Radiation Therapy Oncology Group (RTOG) grading scale. All patients completed treatment without interruption. No grade 3 or higher events were observed. Some patients (60%) experienced grade 1 acute toxicity, characterized by mild erythema or dry desquamation, while others (40%) presented grade 2 side effects, mainly moderate erythema or patchy moist desquamation within the treated area.

In the example case involving the hybrid intracavitary-contact approach, shown in Figure 1, the Freiburg flap applicator was successfully adapted to the concave auricular geometry and the inner cavity of the ear. The resulting dose distribution confirmed both the feasibility of the implant and adequate dosimetric coverage of the target: despite the challenging anatomy, V95% was 98.1%, while V150% was 0.3% (0.004 cm^3^), remaining within clinically acceptable thresholds.

## 4. Discussion

The face is anatomically divided into different zones based on the risk of cancer relapse after primary treatment, with areas categorized as high, intermediate, or low risk. The ear, specifically the auricle and adjacent skin, falls within the so-called H-zone, which is considered a high-risk area for skin cancers due to its complex anatomy and proximity to critical structures [11]. Non-melanoma skin neoplasms occurring in the head and neck region account for a significant proportion of skin cancers, with lesions around the ear representing approximately 13–15% of these cases. The auricular region poses significant challenges in surgical management, often leading to difficulties in achieving clear margins and maintaining satisfactory cosmetic outcomes. The presence of positive surgical margins in these cases is a notable concern, as it is associated with high recurrence rates, reported to range between 10% and 67% [12].

Given these challenges, alternative or adjunctive treatments such as IRT have gained importance in managing peri-auricular skin cancers. In a retrospective analysis conducted by Bilski et al., 33 patients with non-melanoma skin cancers of the outer ear were treated using HDR contact IRT. Of these patients, 43% underwent IRT as definitive treatment, while 57% received it as adjuvant therapy due to positive or close surgical margins. The prescribed fraction doses varied between 3 and 7 Gy per session, delivered over a period ranging from 1 to 42 days, with total doses spanning from 7 to 49 Gy. The median follow-up time was approximately 30 months, allowing for meaningful assessment of both efficacy and toxicity outcomes [12].

Acute skin reactions with contact HDR-IRT appear frequent yet generally mild, aligning with expected radiobiologic responses for superficial treatments. These skin toxicities were significantly correlated with the volume of skin receiving a high radiation dose, specifically those receiving 200% of the prescribed dose. Patients who experienced these reactions were more likely to have advanced-stage disease and to have initiated IRT sooner after surgery. Nevertheless, these adverse effects were considered acceptable in the clinical context, and importantly, no severe or late toxicities, defined as CTCAE or RTOG grade III or higher, were reported. This suggests that while contact HDR IRT can lead to manageable skin reactions, its safety profile remains favorable [12].

Overall, HDR contact IRT was demonstrated to be an effective and well-tolerated treatment modality for non-melanoma skin cancers of the peri-auricular region. One of the major advantages highlighted by the authors is the ability to deliver treatment in an outpatient setting, reducing patient burden and logistical challenges compared to more invasive or hospital-based therapies. This outpatient feasibility makes contact IRT an attractive option, especially for patients who are not candidates for surgery or more invasive treatments [12].

In addition to its primary use, IRT may also serve as a boost following external beam radiotherapy (EBRT) in select cases of external ear carcinoma. Clinical results from such combined approaches have been encouraging, showing good tumor control and acceptable toxicity profiles. For instance, Budrukkar et al. and Singh et al. reported favorable outcomes using HDR IRT boost in this setting, supporting its role as a valuable adjunct to EBRT [13,14].

Furthermore, in anatomically complex cases where lesions extend into difficult-to-treat areas such as the external auditory canal, a combined IRT approach using a mold for contact treatment together with an intracavitary technique has been described. In contact IRT, the accuracy of applicator positioning is of paramount importance to ensure optimal dose delivery. The applicator must remain in close apposition to the skin surface, with moderate pressure applied to minimize the risk of air gaps while avoiding excessive compression that could lead to tissue hypoxia or dose inhomogeneity [15,16]. Even small air gaps can theoretically alter the superficial dose distribution, raising concerns about underdosage in the clinical target volume.

Recent technological advances, particularly the use of three-dimensional (3D) printing, have facilitated the fabrication of patient-specific applicators with excellent conformity to complex anatomical surfaces. Indeed, studies have shown that with 3D-printed molds the average error in air-gap dimensions is only 0.4 mm, with a standard deviation of 0.5 mm [6]. This high degree of accuracy suggests that customized 3D-printed applicators can reduce the variability associated with conventional placement techniques, offering a more reliable contact geometry.

Among commercially available solutions, the Freiburg™ flap has long been employed in surface IRT due to its favorable dosimetric characteristics. Its design, composed of small spheres or “pellets,” ensures that scatter effects and interpellet air gaps remain below 5% [17]. Monte Carlo–based evaluations have further confirmed that potential air gaps between the Freiburg™ flap and the skin surface do not result in clinically significant deviations in dose distribution for 192Ir sources [18]. Nevertheless, the possibility of residual air pockets has led some authors to argue in favor of external beam radiotherapy techniques such as VMAT, citing the theoretical concern of dose perturbation [19].

The clinical significance of air gaps appears to be dependent on their size and the treatment modality considered. For instance, in external beam radiotherapy an unwanted gap of 4–10 mm has been reported to reduce the superficial dose by approximately 4–10% [20]. In contrast, IRT applicators demonstrate greater robustness to this issue. Monte Carlo simulations performed with the Leipzig and Valencia applicators modeled flat and irregular surfaces, including an “ear phantom” with multiple gaps. These studies showed that scatter within the applicator itself contributes significantly to dose deposition, thereby mitigating the effect of small gaps. For air cavities up to 5 mm, the observed perturbations were minimal, remaining below 3% on the air-gap side and below 1% on the tissue-contact side [21]. These results emphasize that while larger voids can compromise target coverage, smaller gaps are unlikely to substantially alter clinical outcomes in contact IRT.

As observed from the results in our series, the global CTV coverage is not globally compromised by the presence of air gaps of different thicknesses between the implant and the patient’s body. Nevertheless, this does not imply that air gaps are clinically irrelevant. As shown in Figure 5, even small local air gaps can produce a significant reduction in the delivered dose, which, if occurring in regions harboring residual disease, may lead to underdosage of the target area and, consequently, an increased risk of local recurrence. Therefore, beyond a global assessment of the dose distribution provided by the implant, it is essential to perform a more detailed local evaluation of air gaps, carefully considering both their thickness and, more importantly, their anatomical location. The spatial position of these gaps relative to the CTV plays a pivotal role in determining their dosimetric impact, and their presence should be closely assessed during treatment planning and quality assurance procedures.

Figure 5 shows that the air-gap thickness has the largest influence on skin dose, with increasing distances between the source and the skin producing a marked and progressive reduction in the delivered dose. In contrast, the effect of alginate thickness is milder, resulting in only minor variations in skin dose across the investigated range. These findings emphasize the importance of minimizing unintended air gaps during treatment setup, whereas the influence of alginate thickness is less critical for overall dose distribution.

This study highlights the necessity of considering both anatomical geometry and material properties in contact IRT, particularly in regions characterized by irregular surfaces such as the ear. While the use of conformable materials like alginate can improve implant stability and reduce the occurrence of dose deficiencies, the presence of unrecognized air gaps can still compromise the local dose delivery and should not be underestimated.

By applying the TG-186 formalism, which accounts for heterogeneities in tissue and material composition, we achieved a more accurate simulation of the real physical behavior of alginate and air compared to the TG-43 model. The results demonstrate that an average density of 1.55 g/cm^3^ is sufficient to describe the dosimetric behavior of commercially available alginate used in clinical molds. Furthermore, the limited differences in calculated dose parameters between high- and low-density assumptions support the robustness of this modeling approach, enabling its straightforward implementation in clinical practice without compromising accuracy. Based on our data, it is reasonable to state that small air pockets up to 1 mm do not globally compromise clinically relevant dosimetric parameters.

## 5. Conclusions

This study demonstrated that, in contact HDR-IRT for auricular non-melanoma skin cancer, the presence of small air pockets up to 1 mm does not globally compromise clinically relevant dosimetric parameters. Target coverage (V150%, V100%, V95%) remained comparable between TG-43 and TG-186, while the latter predicted lower D2cc values for the ipsilateral eye, providing a more reliable assessment of OAR exposure. However, in correspondence with air pockets, a local drop in dose can be significant; therefore, particular attention should be paid to ensuring that the mold adheres as much as possible and that air pockets do not occur directly over the target.

## Figures and Tables

**Table 1:** Main clinical findings.

Feature	Value	
Age	Mean 78 (range 62–90)	
M/F ratio	90%/10%	
IRT schedule	3 Gy × 18	30%
	5 Gy × 8	40%
	2.5 Gy × 20	30%
Clinical setting	Exclusive	70%
	Post-operative	30%
Ear side	Right	60%
	Left	40%
Histological type	SCC	70%
	BCC	30%
n. of catheters used	Mean 6 ± 2	
Type of implant	Contact (90%), Contact + intracavitary (10%)	

**Table 2:** Dosimetric comparison between TG-43 and TG-186 formalisms for coverage and dosimetric parameters. Values are expressed as mean ± SD.

	TG-43	TG-186	*p*-Value
V150% CTV (%) ± SD	2.6 ± 2.6	2.5 ± 2.5	0.37
V100% CTV (%) ± SD	89.6 ± 3.6	86.7 ± 6.1	0.28
V95% CTV (%) ± SD	96.2 ± 1.9	94.4 ± 3.5	0.13
D2cc Eye (%) ± SD	4.0 ± 2.0	3.2 ± 2.3	0.08

## Data Availability

The data presented in this study are clinical and are stored within the hospital’s internal information systems.
